# Development of the Hearing Rehabilitation for Older Adults (HeRO) Healthcare Mobile Application and Its Likely Utility for Elderly Users

**DOI:** 10.3390/ijerph17113998

**Published:** 2020-06-04

**Authors:** Chanbeom Kwak, Saea Kim, Sunghwa You, Woojae Han

**Affiliations:** 1 Laboratory of Hearing and Technology, Research Institute of Audiology and Speech Pathology, College of Natural Sciences, Hallym University, Chuncheon 24252, Korea; cksqja654@gmail.com (C.K.); robeiu@naver.com (S.K.); shyouuu@gmail.com (S.Y.); 2 Division of Speech Pathology and Audiology, College of Natural Sciences, Hallym University, Chuncheon 24252, Korea

**Keywords:** digital health, age-related hearing loss, auditory training, technology acceptance model, gerontechnology

## Abstract

The present study aimed to develop a healthcare application for the elderly who suspect or know they have a hearing loss, namely, the Hearing Rehabilitation for Older Adults (HeRO), which is available in a mobile device, and then to confirm its probability of acceptance among elderly users. Under a web server system, HeRO which had four types of tailored training for the aged auditory system (i.e., syllable, sentence, discourse, working memory) and a self-reported questionnaire to screen amount of the hearing loss was completed for the elderly. To verify whether the HeRO contents and functions were user-friendly to the elderly users, the technology acceptance model (TAM) was used. Forty-four older adults were asked to use the developed application for 10 days and then respond to a TAM questionnaire with 25 items. The Cronbach’s α coefficient of each subcategory was very high. The construct validity of all subcategories showed high eigenvalues using principal component analysis. Furthermore, our regression model statistically supported a persuasive intention to use the healthcare application because the elderly readily accept it and find it easy to manipulate. We expect the current technology to be applied to the general public as well as the elderly who want to explore digital health.

## 1. Introduction

Worldwide, the elderly population is rapidly increasing and thus many developed countries have become aging societies. According to the Centers for Disease Control and Prevention (CDC), 34% of older adults develop a poor health condition or disability when they are older than 65 years, and 37% of older adults have three or more chronic diseases when older than age 75 years [1]. Regardless, more than 90% of older adults (over the age of 65) want to live independently [2] and most want to remain in their own home as long as they can [3].

Older adults report that they experience some hearing problems not only in communication with their family and friends, but also in everyday life. Since aging is a main factor that negatively affects an individual’s sensory, perceptual and cognitive functions, older adults’ listening, and communication abilities often deteriorate [4,5,6]. This deteriorated hearing-function of older adults, called “age-related hearing loss” (ARHL), features specific deficits in higher-frequency hearing loss and poor speech recognition in distracting listening environments, such as presence of background noise and reverberation. If ARHL is not treated at the early stage, older people will face decreased cognitive abilities and increased safety risks [7], and their accelerated cognitive decline will result in a high incidence of dementia [8]. Thus, timely and appropriate diagnosis and intervention of hearing loss for older adults should be required to maintain their quality of life [9]. Remarkably, the frequency ranges of hearing loss spread into the middle and lower frequencies with age, simultaneously affecting central auditory processing [10]. As a result, ARHL makes it difficult for older adults to detect and understand certain consonants [11], recognize some sentences [12], and understand fast-rate speech [13], but they can benefit from selective stress on speech [14], which suggests that the elderly require specialized auditory training. In Borella and colleagues’ memory training study, 40 older adults underwent various types of working memory tasks, including forward and backward digit span training for two weeks. Interestingly, the trained group performed significantly better on the forward digit span task than the untrained group [15], which suggests that even older adults who have cognitive decline can benefit from intensive memory training.

On the other hand, aural rehabilitation is an effective intervention for people with hearing loss, regardless of whether they use hearing aids. As a key component of aural rehabilitation, Boothroyd found that auditory training can compensate for hearing deficits and improve the quality of life for people with hearing impairments [9]. In other words, hearing-impaired patients who received systematic and professional auditory training displayed high satisfaction with hearing aids and increased self-confidence. Nevertheless, contemporary researchers have criticized the limitations of traditional auditory training, thus leading to computer-based rehabilitation tools such as computer-assisted speech training (CAST) [16], computer-assisted speech–perception testing and training at the sentence level (CASPERsent) [17], and listening and communication enhancement (LACE) [18]. These newly developed rehabilitation tools are popular among general patients with hearing loss but are not considered for specific populations in an aging society (i.e., the elderly). Moreover, these computer-based rehabilitation tools only provide one-way communication rather than interactive communication, which is more efficient and has better effects. For example, if older adults who wear hearing aids have problems with a training tool or want feedback regarding their training results, they must visit a clinic or hearing center to discuss the situation with the clinician in a face-to-face meeting. To resolve these difficulties for older adults and if considering the increased aging population who live alone or do not want to move, the importance of telehealth, defined as the remote exchange of data between a patient and healthcare professional to assist in the management of an existing long-term condition [19], is deserved to grow.

In public health, technology is generally defined as any electronic or digital product or service for the health [7] and has grown quickly during the past 15 years [20]. Today, technology can even support monitoring, maintaining and managing health-related conditions and diseases (CDC, 2006). The American Association of Retired Persons (AARP) has supported that older adults are aware of the importance of technology and intend to use technology to maintain their independent life [3]. Because various technologies developed for telehealth application can help older adults overcome limitations of time, distance and cost [16,17,18], and thus can be useful in their daily life and in their health care and safety in the aging society [7], the older adults should be persuaded to access new technologies. Mitzner and his colleagues tried to identify the factors that influence older adults’ attitude toward and usage of technology and confirmed that if the technology reflects and focuses on the elderly’s deteriorated physical and cognitive abilities, it can compensate for and resolve certain age-related problems [7]. Although understanding the importance of technology, intending to use it, and accepting and adapting it for older adults is necessary [7], most aural rehabilitation tools, unfortunately, have not yet been verified and/or cannot be customized for elderly users, which gives us justification for the present study.

To analyze how older adults respond to new technology, the technology acceptance model (TAM) [21], which demonstrates the multidimensional and complex factors that influence technology adoption, has been employed in numerous studies. Venkatesh and Davis convinced that TAM could lead actual users to the intention to use (IU) new technology and/or systems via two major factors, perceived usefulness (PU) and perceived ease of use (PEOU) [22], which developed from a background of social capital theory (i.e., social trust, institutional trust, and social participation) and social cognitive theory (i.e., system self-efficacy). In short, previous studies that examined the effects of TAM and relevant theories on telehealth tools suggested that older adults had positive perceptions and/or intentions to use digital health systems [19]. However, we do not fully understand these older adults’ difficulty and/or reluctance as special users who may not always accept new technology quickly. In this light, the present study aimed to introduce the developmental process and contents of a healthcare mobile application-based aural rehabilitation tool, namely, Hearing Rehabilitation for Older Adults (HeRO), and to confirm through a TAM questionnaire whether the application is useful for the elderly.

## 2. Materials and Methods

The study was designed as two phases: In the first phase, we developed the hearing rehabilitation application for launching on the mobile device. The second phase focused on implications of the HeRO application for elderly users.

### 2.1. Phase 1. Development of E-Health Technology

#### 2.1.1. Hearing Screening Tool in HeRO Application

The self-assessment for hearing screening of the elderly—revised (SHSE-R) was used to determine participants’ extent of hearing loss [23]. This questionnaire was developed and standardized while considering the nature of ARHL. It consists of 20 questions in three categories (general issues, distracting conditions and working memory) regarding problems common among the elderly [23,24]. Since the SHSE-R was administered at the beginning of the HeRO mobile application, the users could check their current hearing condition by themselves and be guided through further intervention and rehabilitation based on the amount of hearing loss.

#### 2.1.2. Training Contents of HeRO Application

**Figure 1:**
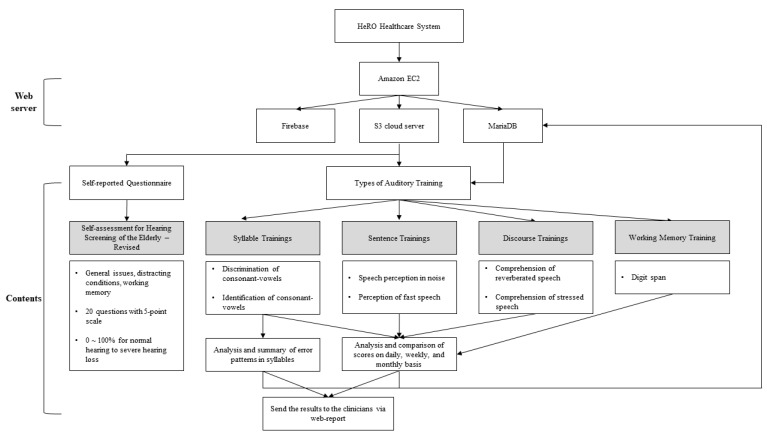
Framework of HeRO healthcare mobile application including web server parts and key contents, which consists of one self-questionnaire and four types of auditory training. It sends a summary of the training results to the clinician and provides feedback to the users (trainees) from the clinician as interactive communication.

The HeRO mobile application offered four types of auditory training. These were featured in seven tools for the elderly (Figure 1). For example, syllable training contained discrimination and identification of consonant–vowel combinations. In sentence training, speech comprehension was intended to train for perception ability in background noise and fast-rate speech with time-compressed sentences. For discourse training, comprehension of reverberated and stressed speech was required to improve perceptual ability in daily circumstances. Finally, working memory training re-organized digits in ascending order when they were randomly presented.

All the training, except for working memory, had three levels of difficulty—easy, moderate and hard. For reverberated speech and selectively stressed word training, short passages on six topics were presented, followed by four questions. The level of difficulty in working memory training was represented by the number of digits presented. All stimuli in the HeRO mobile healthcare application were recorded by a female native speaker of Korean who was a professional announcer.

#### 2.1.3. Stimuli for Self-Training Tools in HeRO Application

##### Syllable Trainings Using Consonant–Vowel Combinations

Syllable training consisted of discrimination and identification of consonant–vowels (CVs). The discrimination training had two stages. The first stage involved presenting three CV words and identifying whether the words were the same or not. In the second stage, the users selected which word was different among three CV words. The identification of CV training was similar to discrimination training, but the users were asked to select a CV word from three words that they heard (at the first stage) or type the word (at the second stage). That is, the first stage of training was closed-set using three examples of CV words, whereas the second stage was open-set and did not present any examples. The easy level was a quiet condition. The moderate and hard levels had a +6 dB and +3 dB signal-to-noise ratio (SNR) with white noise, respectively. The correct percentage of the three levels for the two trainings was calculated by the number of correct syllables among the given syllables in each condition.

##### Sentence Trainings under Background Noise and Fast Rate of Speech

The sentence training included improvement of speech perception in noisy circumstances and enhancement of perception in fast speech. For the sentence with background noise, the training stimuli used the lists from the Korean speech perception in noise (KSPIN) test [25]. While the original KSPIN lists offer random high and low predictability of hitting a keyword in the sentence, we reorganized each of the sentence lists for either high or low predictability for the type of training. The users were required to listen to the sentence with a question tag and type the keyword in the application screen. Quiet, + 6 dB and + 3 dB SNR conditions were allocated as the levels of difficulty. The results showed the percentage that was correct in the same manner as syllable training.

Rapid sentence comprehension training was intended to improve the comprehension of fast speech. The Korean speech audiometry (KSA) sentence list was used as the stimuli [26]. To modulate the speed of the sentence, Adobe Audition 2.0 software (Adobe Systems, Inc., San Jose, CA, USA) was used. The rapid sentence comprehension training required the user to listen to the sentence and then to type as much of the sentence as he or she heard. There were three levels of difficulty: 10%, 30% and 50% modulated time compression. To calculate the correct percentage, each sentence was analyzed based on meaningful and meaningless words. Meaningless words were expletives, and meaningful words were all the other parts of speech. The results for the total number of trainings showed the correct percentage.

##### Discourse Trainings for Comprehension of Reverberated and Stressed Speech

A short discourse with various reverberation circumstances was used for sentence comprehension in reverberation training. This training focused on improvement in listening and comprehension ability in a reverberant situation. The discourse consisted of six topics that were familiar to the elderly: sports, travel, food, common sense, medicine and old age. Each topic had two short discourses with three levels of reverberation time (RT) such as 500, 1500 and 2500 ms manipulated by Adobe Audition. In total, 36 discourses (6 topics × 3 levels of difficulty × 2 discourses) were applied.

The stressed word in sentence training used the selected stressed word within a sentence with various rates of time compression. This training used a short discourse with six different topics. The topics were traditional fairy tale, travel, movie, food, common sense and mystery. The stressed word was chosen as the keyword in the sentence and adjusted to + 6 dB higher in intensity than other components within the sentence. Each 10%, 20% and 30% of modulated time-compression was adjusted to the level of difficulty. The procedure and total number of discourses were the same with sentence comprehension in reverberation training.

##### Working Memory Training Using Digit Span

To improve the working memory of elderly users, a digit span test was used. This training consisted of six levels of difficulty containing digits of various lengths, from three to eight. The user was asked to listen to a series of numbers and list them in ascending order [27]. For example, if the three digits were 8, 2 and 6, the user needed to type 2, 6 and 8. The level of difficulty increased when the participant gave three correct answers in a row. The result counted the longest number that the user could manipulate.

#### 2.2. Phase 2. Evaluation of E-Health Technology

##### 2.2.1. Participants

To identify the acceptance of the HeRO application, 44 elderly people (27 females, 17 males) in local residents participated voluntarily. Their mean age was 72.89 years (SD: 3.88). Most participants had graduated from high school as the minimum education level (9 university graduates, 1 middle school graduate). Before beginning the experiment, hearing screening tests were performed. Having no otological history, no chronic disease such as diabetes and normal function of the middle ear, participants’ hearing fell within the normal range of 250 Hz– 4000 Hz with no air–bone gap as a function of age [28]. In addition, participants showed a normal cognitive function with scores of 25 or higher on the mini mental status examination—Korea (MMSE-K) [29].

All participants were native Korean speakers and signed an informed consent form before the experiment. All procedures were approved by the Institutional Review Board of Hallym University (#HIRB-2017-124).

##### 2.2.2. Data Collection and Analysis

A modified questionnaire based on TAM [21] was used: three subcategories (i.e., PU, PEOU and IU) with 25 items (i.e., 10 items for PU, 10 items for PEOU and 5 items for IU). For each item, a subjective 7-point Likert scale ranging from strongly agree (7) to strongly disagree (1) was employed. The HeRO application was not released yet for any commercial purpose, so that the application was installed to a 10-inch tablet (Galaxy Tab A6, Samsung, Seoul, Korea) using APK file which used in Android operating system. Then, we provided the same tablet to the all participants for 10 days. During the HeRO application usage, 8 of 44 participants who had any kind of problems with the application contacted to the researchers to deal with related issues. After the practice period (minimum recommended time was 30 min to 1 h per day), the individual participants responded to the results. To identify the results of the questionnaire statistically, multiple linear regression analysis was conducted. Before performing multiple linear regression analysis, principal component analysis with varimax rotation was used to re-arrange the items of the questionnaire and to speculate about the insights of the questionnaire results.

## 3. Results

### 3.1. Phase 1. Development of E-Health Technology

#### Structure of the HeRO Application

**Figure 2:**
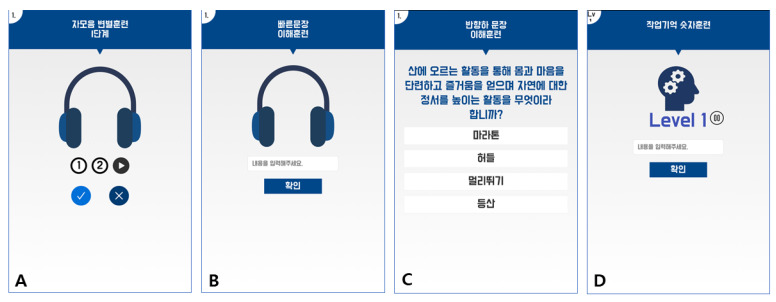
Image examples of the developed HeRO healthcare mobile application for four types of auditory training in the present study: (**A**) syllable training, (**B**) sentence training, (**C**) discourse training and (**D**) working memory.

In development of the HeRO application, five platforms were used. The Amazon EC2 cloud server, which provides high security and scalability, was used to build the application programming interface (API) server. To construct the API server, exchange the data and communicate using Rest API and Client (Android), the Node.js was applied. The PM2 was used to generate and monitor the server. The sound files were saved in the S3 cloud. Because the HeRO application had a sign-up, log-in and client database, the Firebase platform was used. The training database was the MariaDB, which had high stability and reference based on MySQL (see the web server part of Figure 1). Figure 2 provides a sample image of the developed HeRO healthcare mobile application for four types of auditory training.

The application had four specific and useful functions. First, the training results showed the changes in each training result per month. This option can help both user and clinician identify changes in the results and guide the training. Second, to confirm the results, the error pattern was included in the training results. This option showed the error pattern, which gathers all the user’s responses and analyzes the incorrect answers. Third, the results report for the clinician option provided a real-time report of the training result. Fourth, the clinician provides feedback on the results of the user’s training and remotely fits/re-fits the hearing aids users already use.

### 3.2. Phase 2. Evaluation of E-Health Technology

#### 3.2.1. TAM Questionnaire Results

In Table 1, the results of TAM questionnaire were summarized with 44 respondents. With high positive PEOU as 5.86 points out of 7-point scale (item #6), they reported that it was easy for them to remember how to perform tasks using the HeRO application system. In addition, PU of the developed application showed overall high scores between 4.43 and 5.70 points. They believed that to use the application would provide beneficial to their life (item #4 of IU).

In the subcategories of TAM questionnaire, IU items had the highest scores (5.38 points, SD: 0.93) and PEOU and PU items followed at 5.13 points (SD: 1.37) and 5.27 points (SD: 1.15), respectively. Due to some items of PEOU which with negative meaning, items of odd number in PEOU were calculated with inverse coding. While showing high internal consistency, the Cronbach’s α coefficients were 0.923, 0.846 and 0.813 for PU, IU and PEOU, respectively.

While being measured by principal component analysis (PCA) using varimax and a predefined set of three factors, the construct validity was examined. The eigenvalues for the factors were 29.41 for factor 1 (IU), 19.63 for factor 2 (PEOU) and 16.92 for factor 3 (PU), explaining 65.97% of the total variances. Estimations of the three predefined factors were confirmed by a scree plot showing the eigenvalue for each factor. The factor loadings against each item are presented in Table 2. In factor 1 (IU), 12 items (4 each for IU, PEOU and PU) are included. Factor 2 (PEOU) and factor 3 (PU) have 7 items (5 for PEOU and 2 for PU) and 6 items (4 for PU, 1 for IU and 1 for PEOU).

#### 3.2.2. Multiple Regression Model of TAM

**Figure 3:**
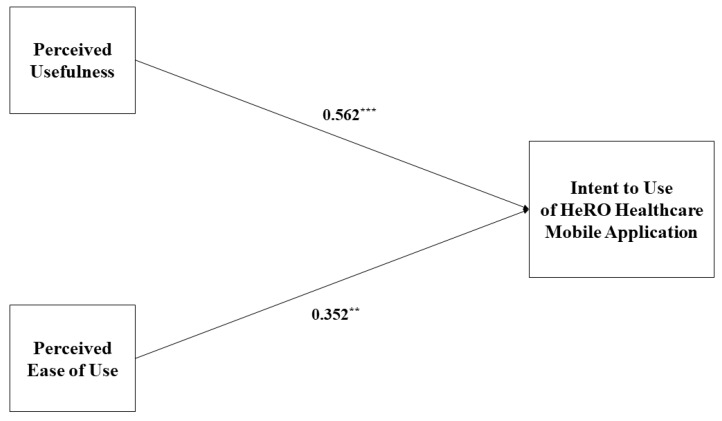
Path analysis of HeRO healthcare mobile application likelihood of use based on multiple regression model. * *p* < 0.05, ** *p* < 0.01, *** *p* < 0.001.

Based on results of the PCA, we re-arranged items of the TAM questionnaire and conducted a multiple regression analysis (see Table 3), which showed 0.672 of adjusted R^2^ (*p* < 0.01). Figure 3 explains that our regression model supports a persuasive relation from PU to IU (0.562, *p* < 0.01) and from PEOU to IU (0.352, *p* < 0.01).

## 4. Discussion

Successful hearing rehabilitation should include four requirements: identifying individual needs, setting specific goals, making shared informed decisions and supporting self-management [30]. Particularly for supporting the self-management, the user can identify their problem, seek appropriate feedback, and receive support from clinicians if necessary. Considering those requirements, the present study aimed to develop the new HeRO healthcare mobile application to provide the best telehealth technology with an effective hearing rehabilitation tool for elderly users.

Historically, Sweetow and Sabes introduced the listening and communication enhancement (LACE) program and tried to observe the interaction between patients with hearing loss and the LACE program [18]. The LACE program was consisted of speech perception tests such as the speech in babble test, time-compressed speech test, competing speaker test, auditory working memory test and speech processing test. Although the LACE program provides supplemental information through the user’s computer, it is important to consider the actual and real-time response to the training results and process for users, instead of saving the data in the home computer system. Our participants who used the HeRO application agreed that it was more convenient than the computer-based program since the program can be installed in their own smartphone as an application, while reporting high values in PEOU. Furthermore, to overcome the limitations of other programs, the HeRO application included the additional context of aural training, such as a self-reported hearing questionnaire and working memory and useful functions that allow timely interaction between user and clinician. In summary, the developed HeRO healthcare mobile application offers the key benefits of time, resource and cost-effective intervention including convenient access by the user without limitation of place, the potential to tailor training packages to individual needs and the ability to remotely monitor and capture user data for the clinician.

Regardless of the user’s age and/or degree of disability, the intervention technology should be effective, identify tangible benefits from the training program and verify the users’ intention to use the training program [7,30,31]. The results of path analysis in the current study demonstrated that older adults are aware of the importance and necessity of health-related technology (e.g., the HeRO) and they showed positive perceptions and willingness to use. Based on the item #1 of PEOU which was “I find the HeRO application system cumbersome to use” and item #4 of PU which was “The HeRO application supports critical aspects of my life” showed 5.09 and 4.61 points, respectively. These results suggest that older adults can easily deal with the HeRO application and recognize it is beneficial in their life. Again, three variables of TAM means that, (1) PEOU, represents how natural it is to operate the HeRO application, (2) PU, refers to the utility and especially the practical worth of the HeRO application and (3) IU, reflects the act or willingness to use the HeRO application. The HeRO application was easy to use, and the elderly accepted it. Participants in the current study were older adults who were inevitably and negatively affected by aging, so that their sensory and cognitive abilities were deteriorated [6]. Mitzner and colleagues suggested that older adults had positive perceptions toward technology and the major factors affecting their positive perceptions were reduced physical and/or mental effort [7]. Put simply, the deteriorated abilities of older adults can be compensated by the use of technology. Also, the authors analyzed their data in various domains such as health, home and work and concluded that specific features (i.e., portability and size, date and time on devices) were significant factors for positive perception about technology, especially in the health domain. Specifically, for health-related technology, the design such as being small and portable was regarded as a key factor.

In a previous study of the computer-based phonemes training tool [31], the three main factors of TAM showed that PEOU and PU were good predictive variables in the multiple regression analysis for the training tool. Yang and Lay partially support the current study in which the PEOU and PU were significant variables to assist the IU. While both PEOU and PU significantly contributed to IU in the regression model, PEOU showed lower adjusted R^2^ than PU. Chen and Chan, who developed a modified version of TAM while focusing on the gerontechnological model [32], reported that aging was a determinant negatively associated with PEOU. While the previous study used various age groups, such as 55 to 64, 65 to 74, 75 to 84 and over the age of 85 years, most participants were 55 to 74 years old (48.1%). Owing to the relatively higher age of participants in the present study compared to the previous study, we may surmise that PEOU was negatively affected by aging. Moreover, based on the results of previous studies [19,32], various determinants affect PEOU such as health conditions, attitude toward life and satisfaction, physical functioning and technological anxiety. Thus, various factors that affect perceptions of and/or intention to use technology for older adults should be considered in future studies to identify the precise effects of digital health tools for older adults.

Despite the beliefs of some, elderly people accepted new technology. Chen and Chan agreed that deteriorated sensory abilities such as hearing, and vision may influence the ease of use of technology because of graphical/text-based or sound-based interfaces [32]. Although older adults in the current study had normal hearing ability as a function of age [28], it is possible that the aging effect may influence the PEOU. However, we unfortunately encountered a barrier in that a few older people refused health technology because they found it difficult to use. In other words, the multiple linear regression model showed that PU had a higher standardized coefficient than PEOU, which demonstrated users’ difficulties. Thus, we tried to determine the reason why PU was regarded as a priority for IU although both PU and PEOU were statistically significant. As one of explanation, PEOU is defined as the degree to which a person believes that using a particular system will be free of effort [21]. In the other words, PU—or the degree to which a person believes that using a particular system will enhance his or her job performance—is significantly and more strongly linked to IU than PEOU, and if it is high toward a particular system, users have a positive use–performance relationship with the system. Limitations of the current study such as absent of the control group and any changes in perception and attitude before/after using the e-health technology warrant further research. Also, it is necessary to confirm the feasibility and connectivity of techniques and applications related to hearing aid. Although the HeRO application recommended to perform all trainings in the sound field environment, the appliance of hearing aid related to high techniques such as wireless Bluetooth connection [33] could be deal with possible issues for the hearing aid users in terms of coupling, (i.e., dual sound processing) in the further study.

## 5. Conclusions

To improve older adults’ communication abilities—and enhance their quality of life—hearing rehabilitation is often necessary. Mizner et al. reported that the use of health-related technology is beneficial in terms of daily living, finances and medical care for older adults [7]. Although older adults are considered to be digitally divided, which refers to a distinction between individuals who are and those who are not willing to use technology [7], most older adults in the current study reported a willingness to adopt and use the HeRO application. The key reason for this willingness and intention is that the HeRO application may help users overcome the constraints of traditional aural rehabilitation. The term “gerontechnology,” which refers to technology that reduces problems and/or difficulties for the elderly that stem from aging and to provide healthy, independent and social communication [34]. The aging populations are spread across the globe and the number of older adults will increase in the coming years. In this perspective, focused technology, including health-related technology, should be considered and developed to meet the needs of older adults. In conclusion, the results of the present study have demonstrated that older adults considered the HeRO application useful in enhancing aural communication. We believe that the HeRO application will demonstrate the benefits of comprehensive rehabilitation in people with ARHL in future studies.

## Figures and Tables

**Table 1:** Group mean scores and standard deviation for original items of technology acceptance model questionnaire.

Category	No.	Question	Mean	SD
Perceived Ease Of Use	1	I find the HeRO application system cumbersome to use.	2.98	1.27
	2	Learning to operate the HeRO application system is easy for me.	5.55	1.62
	3	Interacting with the HeRO application is often frustrating.	3.30	1.21
	4	I find it easy to get the HeRO application system to do what I want it to do.	5.55	1.35
	5	The HeRO application is rigid and inflexible to interact with.	2.61	1.30
	6	It is easy for me to remember how to perform tasks using the HeRO application system.	5.86	1.07
	7	Interacting with the HeRO application requires many mental effort.	4.00	1.18
	8	My interaction with the HeRO application system is clear and understandable.	5.02	1.34
	9	I find it takes many effort to become skillful at using the HeRO application.	2.86	1.27
	10	Overall, I find the HeRO application system easy to use.	4.98	1.21
Perceived Usefulness	1	Using the HeRO application improves the quality of my communication.	5.61	1.02
	2	Using the HeRO application useful in my life.	5.61	1.08
	3	The HeRO application enables me to communicate more naturally.	4.82	0.79
	4	The HeRO application supports critical aspects of my life.	4.61	0.99
	5	Using the HeRO application increases my productivity.	4.43	0.87
	6	Using the HeRO application improves my communication performance.	5.09	0.96
	7	Using the HeRO application allows me to communicate more than would otherwise be possible.	5.59	1.09
	8	Using the HeRO application enhances my effectiveness in communication.	5.55	1.27
	9	Using the HeRO application makes it easier to communicate.	5.70	1.27
	10	Overall, I find the HeRO application system useful in my life.	5.66	1.24
Intention to Use	1	All things considered, my using the HeRO application in my life is good.	5.66	0.75
	2	All things considered, my using the HeRO application in my life is wise.	5.70	1.09
	3	All things considered, I think my using the HeRO application in my life is favorable.	5.84	1.01
	4	All things considered, I think my using the HeRO application in my life is beneficial.	6.00	0.89
	5	All things considered, my using the HeRO application in my life is positive.	5.95	0.86

**Table 2:** Summary of principal component analysis results for the 15 questions (except for item 13).

Item	Factor Loading		
	IU	PEOU	PU
PEOU 10	0.876		
IU 2	0.752		
PEOU 2	0.745		
IU 5	0.744		
PU 9	0.694		
PU 1	0.669		
PEOU 8	0.660		
IU 1	0.620		
PEOU 7	−0.614		
IU 3	0.610		
PU 2	0.609		
PU 3	0.595		
PEOU 1		0.833	
PEOU 5		0.773	
PEOU 6		0.769	
PEOU 4		0.628	
PU 8		0.611	
PEOU 3		0.495	
PU 5		0.461	
PU 4			0.734
PU 6			0.719
PEOU 9			0.625
PU 7			0.582
IU 4			0.553
PU 10			0.523
Cronbach’s α	0.846	0.813	0.923

PEOU—perceived ease of use; IU—intention to use; PU—perceived usefulness.

**Table 3:** Results of multiple regression analysis with regression coefficients and significance test for questionnaire of technology acceptance model.

Factor	Unstandardized Coefficient	Standardized Coefficient	*t-*Value	Significance
PU	0.577	0.562	5.014	0.000
PEOU	0.344	0.352	3.136	0.003

PU—perceived usefulness; PEOU—perceived ease of use.

## Abbreviations

CAST	computer-aided speechreading training
CASPERsent	computer-assisted speech perception training at the sentence level
LACE	listening and communication enhancement
ARHL	age-related hearing loss
TAM	technology acceptance model
PU	perceived usefulness
PEOU	perceived ease of use
IU	intention to use
HeRO	hearing rehabilitation for older adults
SHSE	self-assessment for hearing screening of the elderly
CVs	consonant-vowels
dB	decibel
SNR	signal-to-noise ratio
KSPIN	Korean speech perception in noise
KSA	Korean speech audiometry
RT	reverberation time
API	application programming interfaceapplication programming interface.
